# Emotional and cognitive effects of menopause and hormone replacement therapy

**DOI:** 10.1017/S0033291725102845

**Published:** 2026-01-27

**Authors:** Katharina Zuhlsdorff, Christelle Langley, Richard Bethlehem, Varun Warrier, Rafael Romero Garcia, Barbara J Sahakian

**Affiliations:** 1 University of Cambridge Department of Psychology, UK; 2 University of Cambridge Department of Psychiatry, UK; 3 University of Seville Instituto de Biomedicina de Sevilla Department of Medical Physiology and Biophysics, Spain

**Keywords:** hippocampus, hormone replacement therapy, menopause, mental health, cognition, entorhinal cortex, anterior cingulate cortex

## Abstract

**Background:**

Menopause is a natural physiological process, but its effects on the brain remain poorly understood. In England, approximately 15% of women use hormone-replacement therapy (HRT) to manage menopausal symptoms. However, the psychological benefits of HRT are not well established. This study aims to investigate the impact of menopause and HRT on mental health, cognitive function, and brain structure.

**Methods:**

We analyzed data from nearly 125,000 participants in the UK Biobank to assess associations between menopause, HRT use, and outcomes related to mental health, cognition, and brain morphology. Specifically, we focused on gray matter volumes in the medial temporal lobe (MTL) and anterior cingulate cortex (ACC).

**Results:**

Menopause was associated with increased levels of anxiety, depression, and sleep difficulties. Women using HRT reported greater mental health challenges than post-menopausal women not using HRT. Post-hoc analyses revealed that women prescribed HRT had higher levels of pre-existing mental health symptoms. In terms of brain structure, MTL and ACC volumes were smaller in post-menopausal women compared to pre-menopausal women, with the lowest volumes observed in the HRT group.

**Conclusions:**

Our findings suggest that menopause is linked to adverse mental health outcomes and reductions in gray matter volume in key brain regions. The use of HRT does not appear to mitigate these effects and may be associated with more pronounced mental health challenges, potentially due to underlying baseline differences. These results have important implications for understanding the neurobiological effects of HRT and highlighting the unmet need for addressing mental health problems during menopause.

## Introduction

Menopause is a key period in a woman’s life during which extensive physical and psychological changes take place, including hot flashes, low mood, and sleep problems (Crandall, Mehta, & Manson, 2023). The menopausal period has been shown to be associated with cognitive decline, such as memory, attention, and language deficits (Greendale, Karlamangla, & Maki, 2020; Schaafsma, Homewood, & Taylor, 2010). Hormone-replacement therapy (HRT) is increasingly being used to minimize menopausal symptoms through replacing ovarian hormones (Bennett & Mathur, 2024). In England alone, the percentage of women prescribed HRT has increased from 11% in 2021 to 15% in 2023 (Public Health England, 2023). According to the NICE guidelines, HRT may be considered to relieve menopausal symptoms, such as depressive symptoms and sleep problems (National Institute for Health and Care Excellence [NICE], 2015, last updated 2024). One systematic review has found an increased risk of depressive symptoms and diagnoses in peri-menopausal women but no difference between pre- and post-menopausal women (Badawy, Spector, Li, & Desai, 2024). While there is evidence from a large randomized controlled trial in 875 women that HRT is effective for the vasomotor symptoms of the menopause (Greendale et al., 1998), there is a limited understanding of the effects of menopause and subsequent HRT use on the brain, cognition, and mental health.

Studies have found contrasting effects of lifetime estrogen exposure and cognitive decline. For example, it has been reported in a prospective cohort study that in women who have had a hysterectomy or an early menopause, dementia risk was increased (Gilsanz et al., 2019). A systematic literature review, on the other hand, did not find an association between exposure to gonadal steroids and dementia, but instead suggested improved cognitive performance and delayed cognitive decline with longer exposure to these hormones (Georgakis et al., 2016). Similarly, contrasting findings have been reported in relation to HRT treatment, with some studies reporting increased dementia risk (Pourhadi et al., 2023; Shumaker et al., 2003) and others showing protective effects of HRT (Kim et al., 2022; Leblanc, Janowsky, Chan, & Nelson, 2001).

Several studies have attempted to untangle how menopause and HRT affect cognition but only few large-scale studies that have investigated how mental health measures are impacted. It has been suggested that both anxiety and depression increase in prevalence in post-menopausal women (Alblooshi, Taylor, & Gill, 2023). Two population-based studies conducted in Finland reported worse mental health in post-menopausal women on HRT compared with those who were not prescribed HRT (Toffol, Heikinheimo, & Partonen, 2013). Sleep disorders are also more prevalent during the post-menopausal period (Li et al., 2021; Tandon et al., 2022). HRT has been reported to improve sleep quality, specifically in women that reported vasomotor symptoms (Cintron et al., 2017), but the effects on the general female population are still unknown. Overall, the impact of mental health after menopause and HRT use have not yet been systematically evaluated in a large cohort. It is particularly important to examine sleep, cognition, and mood, since women during menopause report a high percentage of symptoms in these domains (Monteleone et al., 2018; Porter et al., 1996).

Given our poor understanding of the consequences of menopause as well as the increasing use of HRT, this study set out to investigate how they affect measures associated with women’s mental health, cognition, and brain structure. Specifically, we focus on measures of anxiety and depression as these are the two most common mental health disorders (World Health Organisation, 2025) and are known to be affected by menopause and HRT (Bromberger et al., 2013; Wium-Andersen et al., 2022). We analyze UK Biobank data from nearly 125,000 women classified into three categories: pre-menopause, post-menopause who have never used HRT, or post-menopause who have used HRT. We investigate measures of sleep duration, levels of insomnia and tiredness and whether they are mitigated by HRT. We also assess if mood differs between groups by focusing on anxiety and depression measures. Finally, given that previous studies found that menopause and HRT influence cognition, we examine how performance on memory tasks is affected. In the subset of individuals with imaging data available (*n* = 10,873), we assess hippocampal gray matter volumes due to their role in memory, with gray matter loss known to be associated with cognitive decline (Van De Mortel, Thomas, & Van Wingen, 2021). Gray matter volumes from the anterior cingulate cortex (ACC) are also investigated, as it is an area involved in emotional regulation and cognitive control and is altered in mood disorders (Webb, Weber, Mundy, & Killgore, 2014). We hypothesize that measures of cognition and mental health worsen because of menopause but are improved following HRT use due to the replenishment of ovarian hormones. Our analyses provide an extensive overview and quantification of the impact of menopause and HRT use.

## Methods

### Study population

The UK Biobank is a large cohort study including 502,486 men and women who have been recruited in the United Kingdom between 2006 and 2010. The participants were aged 40–69 at recruitment to investigate risk factors for diseases of middle and old age. Data for all participants were collected at multiple timepoints and included questionnaires, cognitive tasks, and MRI scans. Ethics approval was given by the North West Multicentre Research Ethics Committee. Written consent was provided by all participants before starting the study. Data access can be requested from the UK Biobank.

**Figure 1.**
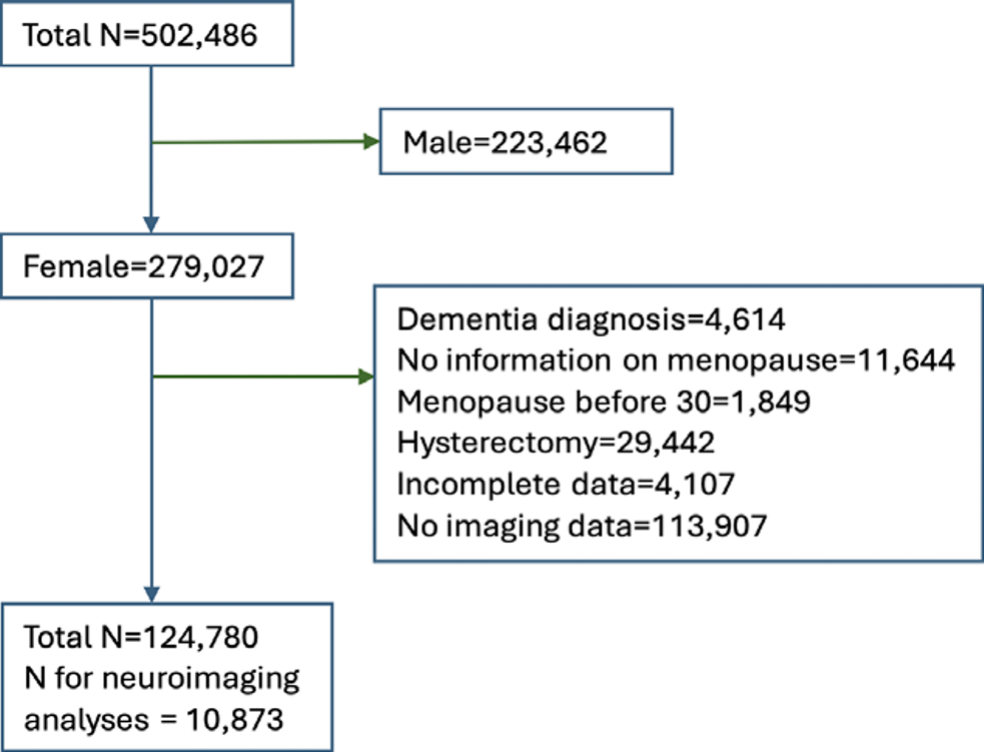
Participant selection flowchart.

From the initial sample of 502,486 participants, only women were included. Following exclusion of participants with a diagnosis of dementia according to ICD10 classification at the first assessment, women who have previously had a hysterectomy, women with age of menopause onset before the age of 30, and participants with incomplete data, 124,780 female participants remained for further analysis (Figure 1).

The pre-registration for our study can be found at the Open Science Framework (https://doi.org/10.17605/OSF.IO/WHU4J).

### Data selection

A touchscreen questionnaire was used to collect information from participants. The following information from these questionnaires was used in the present analysis:

1. Menopause status (Data-Field 2724) – ‘Have you had your menopause (periods stopped)?’. Possible answers: Yes/No/Not sure – had a hysterectomy/Not sure – other reason.
2. Age at menopause (Data-Field 3581) – ‘How old were you when your periods stopped?’. Participants could enter a number, or press ‘do not know’ or ‘prefer not to answer’.

Time since menopause onset was then determined by subtracting the age at menopause from the age at assessment.

1. HRT status (Data-Field 2814) – ‘Have you ever used hormone replacement therapy (HRT)?’. Possible answers: Yes/No/Do not know/Prefer not to answer.
2. Age started hormone-replacement therapy (HRT) (Data-Field 3536) – ‘How old were you when you first used HRT?’ Participants could enter a number, or press ‘do not know’ or ‘prefer not to answer’.

Duration of HRT use was determined by subtracting the age that HRT was started at from the age at assessment.

1. Frequency of depressed mood in the last two weeks (Data-Field 2050) – ‘Over the past two weeks, how often have you felt down, depressed or hopeless?’

Possible answers were: Not at all/Several days/More than half the days/Nearly every day/Do not know/Prefer not to answer.

1. Frequency of unenthusiasm/disinterest in the last two weeks (Data-Field 2060) – ‘Over the past two weeks, how often have you had little interest or pleasure in doing things?’

Possible answers were: Not at all/Several days/More than half the days/Nearly every day/Do not know/Prefer not to answer.

1. Frequency of tenseness/restlessness in the last two weeks (Data-Field 2070) – ‘Over the past two weeks, how often have you felt tense, fidgety or restless?’
  Possible answers were: Not at all/Several days/More than half the days/Nearly every day/Do not know/Prefer not to answer.
2. Self-reported depression/anxiety (Data-Fields 2090 and 2100) – ‘Have you ever seen a general practitioner (GP) for nerves, anxiety, tension or depression?’ and ‘Have you ever seen a psychiatrist for nerves, anxiety, tension or depression?’, respectively. Possible answers were: Yes/No/Do not know/Prefer not to answer.
3. Sleep duration (Data-Field 1160) – ‘About how many hours sleep do you get in every 24 hours? (please include naps)’. Participants could enter a number, or press ‘Do not know’ or ‘Prefer not to answer’.
4. Insomnia (Data-Field 1200) – ‘Do you have trouble falling asleep at night or do you wake up in the middle of the night?’. Possible answers: Never, rarely, Sometimes, Usually, Prefer not to answer.
5. Tiredness (Data-Field 2080) – ‘Over the past two weeks, how often have you felt tired or had little energy?’. Possible answers: Not at all, Several days, More than half the days, Nearly every day, Do not know, Prefer not to answer.
6. Household income (Data-Field 738) – ‘What is the average total income before tax received by your HOUSEHOLD?’. Possible answers: Less than £18,000, £18,000–£30,999, £31,000–£51,999, £52,000 to £1000,000, Greater than £100,000.
7. Qualifications (Data-Field 6138) – ‘Which of the following qualifications do you have? (You can select more than one)’. Possible answers: College or University degree, A levels/AS levels or equivalent, O levels/GCSEs or equivalent, CSEs or equivalent, NVQ or HND or HNC or equivalent, Other professional qualifications, e.g. nursing or teaching, None of the above, Prefer not to answer.

The highest level of education was used for subsequent analyses.

1. Smoking status (Data-Field 20116) – ‘Do you smoke tobacco now?’. Possible answers: Yes on most or all days, Only occasionally, No, Prefer not to answer.
2. Antidepressant/anxiolytic use (Data-Field 20003) – ‘Do you regularly take any other prescription medications? (Do not forget medications such as puffers or patches)’. Possible answers: Yes, No, Do not know, Prefer not to answer. If participants answered ‘yes’, they were followed up on this in a verbal interview. If participants indicated they took citalopram, escitalopram, fluoxetine, fluvoxamine, paroxetine, sertraline, alprazolam, clonazepam, diazepam, lorazepam, or oxazepam, they were marked as taking antidepressant/anxiolytic medication.

The Mental Health Questionnaire asked participants the following questions: ‘Over the last two weeks, how often have you been bothered by any of the following problems?’

1. Recent easy annoyance or irritability (Data-Field 20505) – ‘Becoming easily annoyed or irritable’
2. Recent feelings or nervousness or anxiety (Data-Field 20506) – ‘Feeling nervous, anxious or on edge’
3. Recent inability to stop or control worrying (Data-Field 20509) – ‘Not being able to stop or control worrying’
4. Recent feelings of foreboding (Data-Field 20512) – ‘Feeling afraid as if something awful might happen’
5. Recent trouble relaxing (Data-Field 20515) – ‘Trouble relaxing’
6. Recent restlessness (Data-Field 20516) – ‘Being so restless that it is hard to sit still’
7. Recent worrying too much about different things (Data-Fields 20520) – ‘Worrying too much about different things’

Possible answers to questions 16–22 were Not at all/Several days/More than half the days/Nearly every day/Do not know/Prefer not to answer.

A modified Patient Health Questionnaire (PHQ-4) was calculated based on the following Data-Fields: 2050, 2060, 2070 and 2080, based on previous publications (Gao et al., 2023).

A modified General Anxiety Disorder (GAD) score was approximated using the following Mental Health Questionnaire Data-Fields: 20505, 20506, 20509, 20512, 20515, 20516, 20520.

Cognitive measures were also assessed using a touchscreen interface. The prospective memory (Data-Field 20018), digit-span (Data-Field 4282), and processing speed tasks (Data-Field 20023) were designed so that they could be undertaken without supervision.

1. Prospective memory – participants were given the following instructions at the beginning of the task: ‘At the end of the game we will show you four colored symbols and ask you to touch the Blue Square. However, to test your memory, we want you to actually touch the Orange Circle Instead’.

Possible outcomes were instruction not recalled (skipped or incorrect), correct recall on first attempt, and correct recall on second attempt.

1. Digit-span task: Participants had to memorize an increasing number of digits on each trial, starting with two digits, and a maximum of 12 digits. The highest number of digits recalled was recorded.
2. Processing speed – participants were presented with two cards and were told to press a button as quickly as possible when the two cards presented were identical. Twelve pairs of cards were presented. The mean reaction time was used as an outcome measure.

A participant’s age was determined based on the date of birth provided on the day the attended the Assessment Centre (Data-Field 20122). Their BMI was based on height (Data-Field 50) and weight (Data-Field 21002), which were also collected at the Assessment Centre visit. ICD10 diagnoses (Data-Field 41202) were based on the codes recorded during the participants’ hospital inpatient records. Dementia codes used were: F00 – dementia in Alzheimer’s disease, F01 – vascular dementia, F02 – dementia in other diseases classified elsewhere, F03 – unspecified dementia, F04 – organic amnesic syndrome, F04 – delirium, G30 – Alzheimer’s disease, G31 – other degenerative diseases of the nervous system. For a recurrent depressive disorder diagnosis, code F33 was used. For an anxiety disorder diagnosis, code F41 was used. Participants that had missing outcome variables or responded ‘do not know’, ‘not sure’ or ‘prefer not to answer’ were excluded from further analyses.

### MRI acquisition

MRI scans were acquired across three centers with the same scanners (Siemens Skyra 3 T, Siemens Healthcare, Erlangen, Germany) using a 32-channel head coil (Siemens Medical Solutions, Germany). Participants underwent a high-resolution (1 mm isotropic voxel), T1-weighted, 3D magnetization-prepared gradient echo (MPRAGE) structural scan using the following parameters: 1.0 mm isotropic resolution, TR = 2000 ms, TE = 2.01 ms, TI = 880 ms, flip angle = 8 degrees, FOV = 208×256×256 matrix). T2-weighted fluid-attenuated inversion recovery (FLAIR) structural imaging was obtained using the following parameters: 1.0 × 1.0 × 1.1 mm resolution, TR = 5000 ms, TE = 395.0 ms and TI = 1800 ms. Imaging was done during a separate visit in 2014, up to 8 years later than the initial assessment visit (which took place between 2006 and 2010).

### MRI pre-processing

The pre-processing pipeline has previously been published (Warrier et al., 2023). Briefly, minimally processed T1 and T2-FLAIR-weighted data were accessed from the UK Biobank (application 20904). The images were pre-processed with FreeSurfer (v6.0.1) using the T2-FLAIR-weighted image to improve pial surface reconstruction when available (Fischl et al., 2004). Recon-all reconstruction included bias field correction, registration to stereotaxic space, intensity normalization, skull-stripping, and white matter segmentation. A triangular surface tessellation fitted a deformable mesh model onto the white matter volume, providing gray–white and pial surfaces with >160,000 corresponding vertices registered to fsaverage standard space. When no T2-FLA1R image was available, FreeSurfer reconstruction was done using the T1-weighted image only. Cortical surfaces were reconstructed using FreeSurfer and registered using FreeSurfer’s surface-based registration to fsaverage. For further parcellation, the HCP Multi-Modal Parcellation 1.0 brain region atlas was used, which contained 180 cortical regions per hemisphere (Glasser et al., 2016). The parcellation was resampled from fs_LR to fsaverage using existing transformations and then back to the individual’s surface meshes based on the FreeSurfer folding-based surface registration (Van Essen et al., 2012).

For the present analyses, the hippocampus, entorhinal cortex (EC) and anterior cingulate cortex (ACC) were selected. The following brain areas were added to construct gray matter volumes of the ACC based on the HCP atlas: a24, d32, p24, p32, s32 (Glasser et al., 2016). Volumes from left and right hemispheres were merged.

### Data analysis

This analysis was a three-group, unpaired design. The three groups were pre-menopausal women, post-menopausal women on HRT, and post-menopausal women not on HRT. Please see data selection section for more information on how the different groups were determined.

The following outcome variables were investigated: whether or not the participant had seen a GP for symptoms of anxiety, nerves or depression, whether or not the participant had seen a psychiatrist for symptoms of anxiety, nerves or depression, diagnosis of major depressive disorder or anxiety disorder according to the ICD10 classification, sleep duration, insomnia, tiredness, antidepressant/anxiolytic medication use, as well as the following cognitive measures: prospective memory, digit-span, and processing speed. Additionally, gray matter volumes from the following areas were selected: hippocampus, EC, and ACC. The data used were taken from the first participant assessment in this study and the neuroimaging visit.

The following predictors/covariates are included in the statistical models: age, household income, level of education, smoking status, BMI, time since menopause onset, and HRT duration (Barth et al., 2025; Steventon et al., 2023). For gray matter volume, insomnia, sleep duration, tiredness, and cognitive task analyses, a history of depression was also included as a covariate. The neuroimaging analyses also controlled for total intracranial volume (TIV).

Linear models (lm) and generalized linear models (glm) were implemented in R (R Core Team, 2017, version 4.0.4). The latter was used for binary outcome variables, including self-reported depression and anxiety. We had five measures of anxiety and depression. The first two were ‘having seen a GP due to anxiety, nerves or depression’ or ‘having seen a psychiatrist due to anxiety, nerves or depression’ as an indication of the level of depression and anxiety. The third and fourth measures were modified PHQ-4 and GAD-7 scores. The fifth was an ICD 10 diagnoses of depression or anxiety. Subsequent *post-hoc* pairwise comparisons of estimated marginal means with Tukey multiple comparisons adjustment were run using the emmeans package (Lenth et al., 2018; Pinheiro et al., 2021). A *p*-value of 0.05 was set as the threshold to determine statistical significance.

An example of a linear-mixed effects model investigating the effect of menopause on self-reported anxiety is shown below:

Anxiety ~ glm(group + age + time since menopause onset + duration of HRT use + BMI + household income + level of education + smoking status)

## Results

### Sample characteristics

There were a total of 124,780 participants (M_age_ = 53.70 [8.05]) who fulfilled the criteria, with 49,772 women in the pre-menopausal group (M_age_ = 45.88 [3.76]), 52,252 in the post-menopausal group who have never taken HRT (M_age_ = 58.22 [5.46]), and 22,756 in the post-menopausal group who have taken HRT (M_age_ = 60.41 [5.55]). The average age of menopause onset was 49.45 (4.68) and the average age of starting HRT was 49.03 (5.04). This age demographic seems typical of the reported literature on menopause age in the UK, Europe, and the USA (British Menopause Society, 2021; Dratva et al., 2009; Gold et al., 2013). Further baseline characteristics for the sample population are summarized in Table 1.

**Table 1.** Participant characteristics at the initial assessment

Characteristicmean (SD)/*N*(%)	Total (*N* = 124,780)	Pre-menopausal group (*N* = 49,772)	Post-menopausal non-HRT group (*N* = 52,525)	Post-menopausal HRT group (*N* = 22,756)	*p*-value
Age	53.70 (8.05)	45.88 (3.76)	58.22 (5.46)	60.41 (5.55)	<.0001
Age started menopause	NA	NA	50.40 (4.05)	47.26 (5.26)	NA
Age started HRT	NA	NA	NA	49.03 (5.04)	NA
BMI	26.49 (4.96)	26.22 (5.06)	27.77 (5.01)	26.46 (4.60)	<.0001
Higher education	53,222 (42.7%)	22,659 (45.5%)	21,733 (41.4%)	8,830 (38.8%)	<.0001
Annual household income >£30,999	72,089 (57.8%)	36,094 (72.5%)	26,627 (50.69%)	10,368 (45.6%)	<.0001
Current smoker	10,056 (8.05%)	4,728 (9.50%)	3,474 (6.61%)	1,854 (8.11%)	<.0001
Antidepressant/anxiolytic use	2,654 (2.13%)	1,406 (2.82%)	836 (1.59%)	412 (1.81%)	<.0001

### Depression and anxiety

**Figure 2.**
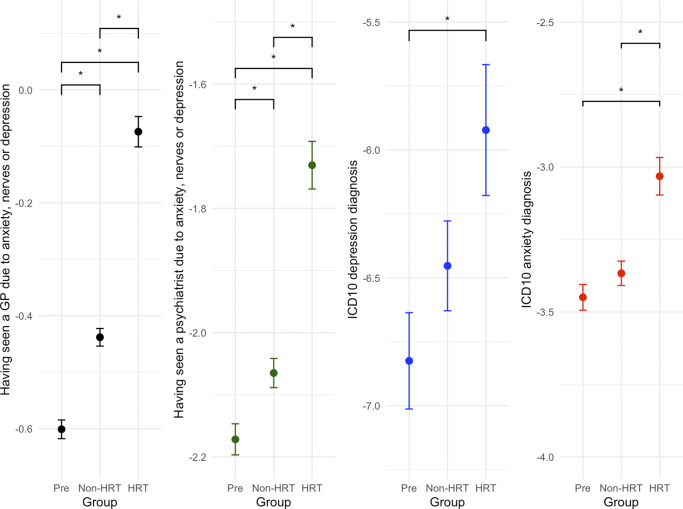
Effects of menopause and HRT on mental health measures: having seen a GP due to anxiety/nerves/depression, having seen a psychiatrist due to anxiety/nerves/depression, a previous ICD10 diagnosis of depression and a previous ICD10 diagnosis of anxiety. All three measures were found to be significantly increased in post-menopausal women (p < .0001). Having seen a psychiatrist or GP due to anxiety/nerves/depression was increased in the HRT compared to the non-HRT group (p < .0001). Y-axis values plotted are the calculated emmeans values for that respective variable.

A significant effect of group on ‘having seen a GP due to anxiety, nerves or depression’ was found ( 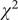 (2,124762) = 263, p < .0001, η^2^p = 0.31) (Figure 2). *Post-hoc* comparisons showed that there were statistically significant differences between all three groups, specifically, both post-menopausal groups reported higher levels compared to the pre-menopausal group (z(124762) = −7.83, p < .0001 and z(124762) = −16.1, p < .0001, respectively). The post-menopausal group on HRT reported higher levels than the post-menopausal group not on HRT (z(124762) = −11.4, p < .0001).

Similar effects on ‘having seen a psychiatrist due to anxiety, nerves or depression’ were found. There were significant group differences in levels of having seen a psychiatrist ( 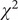 (2,124762) = 84.7, p < .001, η^2^p = 0.054), with the HRT group reporting higher levels than the pre-menopausal group as well as the non-HRT group (z(124762) = −9.37, p < .0001 and z(124762) = −7.42, p < .0001). The non-HRT group also showed higher levels than the pre-menopausal group (z(124762) = −3.32, p = 0.0026).

We combined several self-reported measures to create a metric that aligns with either the PHQ-4 questionnaire or the GAD-7 questionnaire (see Methods). Our modified PHQ-4 score had a significant effect of group (F(2,118281) = 748, p < .0001, η^2^p = 0.01). *Post-hoc* comparisons revealed that this measure was increased in both post-menopausal groups compared to the pre-menopausal group (t(118281) = −10.50, p < .0001 and t(118281) = −13.8, p < .0001, respectively). It was also higher in the HRT compared to the non-HRT group (t(118281) = −7.39, p < .0001). The GAD-7 score showed a similar trend, with a significant group effect (F(2,49971) = c287, p < .0001). Pairwise comparisons showed that the post-menopausal HRT group had higher GAD-7 scores compared to both the pre-menopausal and post-menopausal group not on HRT (t(49971) = −2.80, p = 0.014 and t(49971) = −4.17, p = .0001, respectively).

There was a significant effect of group on ICD10 diagnosis of recurrent depressive disorder ( 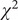 (2,124762) = 8.52, p = 0.014, η^2^p = 2.3e−5). *Post-hoc* analyses showed that there were higher levels of depression in the post-menopausal HRT group compared to the pre-menopausal group (z(124762) = −2.97, p = 0.0084). A significant effect of group on ICD10 diagnosis of an anxiety disorder was also found ( 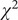 (2,124762) = 27.2, p < .0001, η^2^p = 0.0072). The post-menopausal group on HRT had higher levels of anxiety than both the post-menopausal group not on HRT (z(124762) = −4.35, p < .0001) and the pre-menopausal group (z(124762) = −5.30, p < .0001).

### Anti-depressant and anxiolytic medication use

We assessed whether the three groups differed in terms of antidepressant or anxiolytic medication use. Indeed, we found a significant effect of group ( 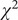 (2,124762) = 20.96, p < .0001, η^2^p = 0.0028). Further *post-hoc* analyses showed that both post-menopausal groups were more likely to be prescribed either anxiolytic or antidepressant medication (pre-menopausal vs non-HRT group: z(124762) = −3.58, p = 0.001 and pre-menopausal vs HRT group: z(124762) = −3.90, p = 0.003). There was no significant difference between the non-HRT and the HRT group.

### Tiredness and sleep

**Figure 3.**
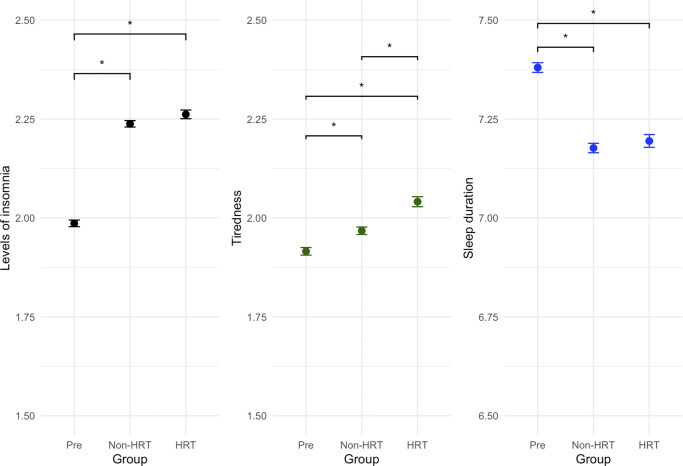
Effects of menopause and HRT on wellbeing measures: levels of insomnia, tiredness and sleep duration. All three measures were found to be significantly increased in post-menopausal women (p < .0001). Tiredness was also found to be higher in the HRT group compared to the non-HRT group (p < .0001). Y-axis values plotted are the calculated emmeans values for that respective variable.

We investigated the effects of menopause and HRT on levels of tiredness, levels of insomnia, and sleep duration. All three measures showed a significant effect of group (tiredness: F(2,124761) = 153, p < .0001, η^2^p = 8.8e-4; insomnia: F(2,124762) = 803, p < .0001, η^2^p = 0.012; sleep duration: F(2,124761) = 224, p < .0001, η^2^p = 0.0034) (Figure 3). For levels of insomnia, the HRT group and the non-HRT group showed higher levels than the pre-menopausal group ((t(124761) = −25.0, p < .0001); (t(124761) = −36.6, p < .0001)), respectively.

The HRT group had higher levels of tiredness compared to the other two groups (HRT vs pre-menopausal: t(124762) = −15.2, p < .0001, HRT vs non-HRT: t(124762) = −14.7, p < .0001). The non-HRT group also showed higher levels than the pre-menopausal group (t(124762) = −6.06, p < .0001).

Sleep duration was decreased in both post-menopausal groups (pre-menopausal vs non-HRT: t(124761) = 20.2, p < .0001, pre-menopausal vs HRT: t(124761) = −11.5, p < .0001), with no differences seen between the HRT and no-HRT groups.

### Cognition

**Figure 4.**
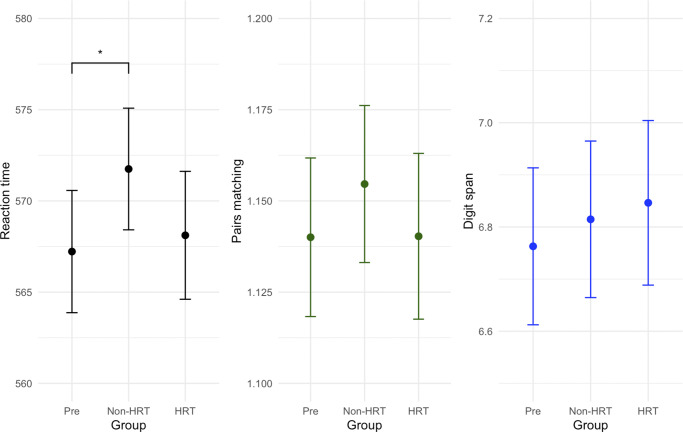
Effects of menopause and HRT on cognitive measures: mean reaction time, prospective memory and digit span. No significant differences between pre- and post-menopausal women and women not on HRT vs those on HRT were found, except slower reaction times in the HRT group vs the pre-menopausal group (p < .0001). Y-axis values plotted are the calculated emmeans values for that respective variable.

The sample size for reaction time was reduced to 124,278 participants as not all participants completed this task. The reaction time was found to be significantly affected by the group (F(2,124259) = 4600, p < .0001, η^2^p = 1.7e−4). *Post-hoc* comparisons showed that the reaction time was slower in the non-HRT group compared to the pre-menopausal group (t(124259) = −4.47, p < .0001), with no other comparisons reaching significance (Figure 4).

For prospective memory, the sample size was reduced to 43,579 participants due to not all participants completing this task. It was found that the group a participant was in (pre-menopause, non-HRT, and HRT) significantly affected performance on this task (F(2,43560) = 27.4, p < .0001, η^2^p = 1.2e−4); however, no *post-hoc* results reached significance following adjustments for multiple comparisons.

The sample size when performing the analysis for the digit span task was reduced to 13,144 participants, as not all participants completed this task. Performance was also affected by group (F(2,13125) = 13.5, p < .0001, η^2^p = 1.4e−4). No *post-hoc* results survived correction for multiple comparisons. Tables with sample characteristics for all three analyses can be found in the Supplementary Materials.

### Brain structure

For the neuroimaging analyses, 10,873 participants were included, as only a subset of participants attended the neuroimaging visit. Of this group, 762 participants were pre-menopausal, 7,583 participants were post-menopausal but have not been on HRT and 2,528 participants were post-menopausal but have taken HRT. The average age of this sample was 63.53 (7.22). A table summarizing the characteristics of this sample can be found in the Supplementary Materials.

**Figure 5.**
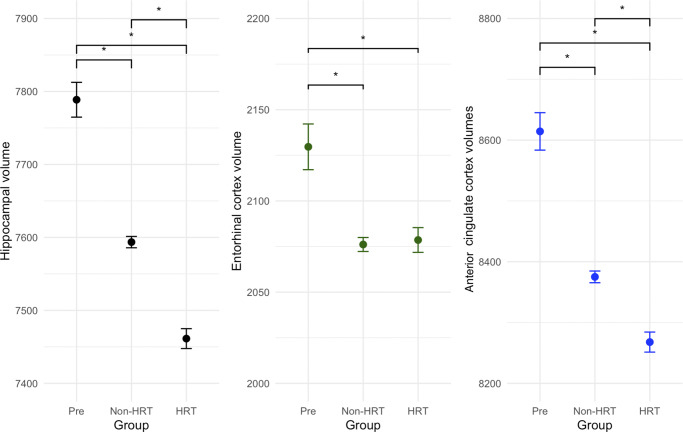
Effects of menopause and HRT on brain volumes: hippocampus, entorhinal cortex and anterior cingulate cortex. All three measures were found to be significantly decreased in post-menopausal women (p < .0001). Volumes were also decreased in the HRT group compared to the non-HRT group in the hippocampus and ACC (p < .0001). Y-axis values plotted are the calculated emmeans values for that respective variable.

There was a significant effect of group on hippocampal, EC and ACC gray matter volumes (F(2,9218) = 88.1, p < .0001, η^2^p = 0.0053; F(2,9214) = 9.82, p < .0001, η^2^p = 4.2e−4; and F(2,9214) = 70.39, p < .0001, η^2^p = 3.4e−4, respectively) (Figure 5). Hippocampal gray matter volumes were significantly decreased in both post-menopausal groups compared to the pre-menopausal group (non-HRT vs pre-menopausal: t(9218) = −6.75, p < .0001 and HRT vs pre-menopausal: t(9218) = −10.6, p < .0001, respectively). Hippocampal gray matter volumes were also reduced in the HRT group compared to the non-HRT group (t(9218) = −7.63, p < .0001).

EC gray matter volumes were reduced in both post-menopausal groups (non-HRT vs pre-menopausal: t(9214) = −3.67, p < .0001 and HRT vs pre-menopausal: t(9214) = −3.31, p = .0002, respectively).

ACC volumes were also significantly decreased in both post-menopausal groups compared to the pre-menopausal group (non-HRT vs pre-menopausal: t(9214) = −7.55, p < .0001 and HRT vs pre-menopausal: t(9214) = −9.87, p < .0001, respectively). ACC volumes were also reduced in the HRT group compared to the non-HRT group (t(9214) = −5.17, p < .0001).

### *Post-hoc* analysis to clarify two possible interpretations of the data

Given the results, there are two possible interpretations (1) that women are prescribed HRT due to existing depression and anxiety and (2) that HRT leads to higher levels of depression and anxiety. To attempt to disentangle these two interpretations of the data, we conducted a *post-hoc* analysis to explore depression and anxiety levels in those who were not on HRT at baseline but were later prescribed HRT.

A total of 87,664 women who did not take HRT at the first assessment visit (2006–2010) reported to have started taking HRT at the second assessment visit (2012–2013). A total of 7,039 of those women had complete data at both time points. We investigated whether these women reported worse mental health outcomes at the first time point compared to women who were not prescribed HRT subsequently. Indeed, we found that the measures ‘ever seen a GP for nerves, anxiety, or depression’ and ‘ever seen a psychiatrist for nerves, anxiety, or depression’ both significantly affected whether someone was taking HRT at the second time point (F(5758) = 11.2, p < .0001, η^2^p = 0.0014 and F(5758) = 4.89, p = 0.027, η^2^p = 0.00076, respectively). *Post-hoc* comparisons showed that these two measures were higher in the group who had started taking HRT between the two time points compared to the group who had never taken HRT (t(5758) = −2.82, p = 0.0048 and t(5758) = −2.09, p = 0.037, respectively). This suggests that participants who have psychiatric symptoms are more likely to be prescribed HRT than those who do not report mental health symptoms.

## Discussion

To our knowledge, this is the first study to investigate the effects of menopause and HRT on mental health, cognition, and brain structure in a very large sample of women.

We found that across several measures of cognition and mental health, women are consistently worse off following menopause. This is demonstrated by the increased levels of depression and anxiety and poorer sleep habits in post-menopausal women. These measures are greater in post-menopausal women on HRT compared to post-menopausal women not on HRT. This trend in mental health measures is supported by reduced gray matter volumes in areas of the brain associated with emotion and memory, with decreased volumes in the MTL and ACC in post-menopausal women generally, which are further decreased in women on HRT compared to women not on HRT. Cognitive measures assessing memory were not affected by menopause or HRT, similar to a previous report (Steventon et al., 2023).

A case–control study conducted in Denmark published in 2023 reported that dementia risk is increased following HRT use, with longer durations of therapy associated with higher hazard ratios (Pourhadi et al., 2023). Similar results were found in a cohort study based in Taiwan (Sung et al., 2022). A randomized controlled trial has also reported that estrogen plus progestin therapy increases the risk of dementia and does not prevent cognitive impairment in postmenopausal women (Shumaker et al., 2003). However, it is important to control for depression as it can alter the associations (Shen et al., 2022) and it can increase the risk of dementia itself (Hickey et al., 2023), and this was not controlled for in the Shumaker et al. (2003) study. Importantly, in our study, participants with an ICD10 diagnosis of dementia were excluded. We measured cognition in middle to older aged women and found no effect of menopause or HRT on memory. In measures of reaction time, we found times were slower in the non-HRT compared to the pre-menopausal group, but there was no difference between the pre-menopausal and HRT group. However, we did find that the HRT group reported higher measures of both anxiety and depression. This aligns with a previous study reporting an increased risk of depression in individuals taking systemic HRT, but not local HRT treatment, particularly in the years following initiation of treatment (Wium-Andersen et al., 2022). One study has found only a small improvement in mood following 4 years of HRT use, whereas another has reported improvements in individuals presenting with menopausal symptoms (Gleason et al., 2015; Joffe et al., 2014). Taken together, these findings suggest that differences in the effects of HRT on mood are likely due to various factors, including whether pre-existing mood symptoms are present, route of administration, and the time of measurement.

In addition to the reported effects on sleep and mental health measures, we found that the hippocampus, entorhinal cortex and ACC had decreased volumes following menopause (following adjustments made for age, TIV, and history of depression/anxiety) and HRT use. Related to our findings, a recent study using the UK Biobank dataset found that later menopause onset correlated positively with larger medial temporal lobe (MTL) volumes (Steventon et al., 2023). MTL areas and the ACC are known to be key areas for mental health and sleep (Brosch et al., 2022; Van Tol et al., 2010), with reduced volumes associated with poorer sleep and psychological measures (Riemann et al., 2007; Shi et al., 2017). Furthermore, a study has reported that hippocampal volumes, memory and sleep are sensitive to loss of estrogen and that reduced volumes relate to disrupted sleep in women with surgically induced menopause, consistent with our findings (Gervais et al., 2023).

It should be noted that in our cross-sectional study, it is difficult to separate whether women who go on HRT have poorer mental health before they are prescribed the medication, or whether their mental health worsens because of it. That is, it is possible that symptoms of anxiety and depression led the women to see their GP in the first place. Our *post-hoc* analysis indicated that it may be the former and that women with pre-existing mental health problems are more likely to be prescribed HRT, suggesting that they may have had poorer mental health at baseline. This interpretation is supported by the fact that the UK prescription guidelines state that HRT should be considered to alleviate depressive symptoms that arise as a result of menopause (National Institute for Health and Care Excellence [NICE], 2015, last updated 2024). However, HRT is not considered to be effective in the treatment of anxiety or depression. While our results suggest that depression and anxiety levels are higher in post-menopausal women on HRT than in post-menopausal women not on HRT, it is uncertain whether these symptoms would be more severe without HRT.

A limitation of the study is that UK Biobank participants tend to be healthier and less ethnically diverse than the general UK population. Furthermore, pre- vs post-menopausal groups were not separated based on STRAW criteria, which would have provided more accurate classification. Instead, self-report measures were used for grouping participants as well as measuring levels of insomnia, anxiety and depression. Data were collected at baseline as to whether they had seen a GP or psychiatrist, however, the date was not specified. Additionally, the UK Biobank dataset did not contain information on the type of HRT participants were taking. It is also important to note that the HRT group had lower levels of higher education, lower income and an older age. Although we have corrected for these variables in our analyses, it is possible that these variables may influence the results. Furthermore, future studies may wish to explore how the male brain changes during the age period corresponding to the menopausal transition in women, as sex-dependent changes in ageing have been reported, although this was beyond the scope of the current work (Bethlehem et al., 2022; Than et al., 2021).

This study provides insight into poorly understood questions regarding menopause and women’s mental health, including the symptoms, such as sleep disturbances, memory problems and psychological symptoms, that menopausal women report the most in surveys and experience as particularly problematic (Huang et al., 2023). Future studies will be able to build on this and aim to identify other factors, e.g. genetic predisposition or co-morbidities (Huh et al., 2024; Saleh, Hornberger, Ritchie, & Minihane, 2023), that could impact how HRT affects the brain and mental health. Our results are important because menopause often leads to heightened emotional difficulties and significantly impacts women’s quality of life, self-esteem, and interpersonal relationships (Deeks, 2003; Parish et al., 2019). Our findings highlight the urgent need for better education, support, and treatment options to help women navigate this challenging life stage more effectively.

## Supporting information

10.1017/S0033291725102845.sm001 Zuhlsdorff et al. supplementary material Zuhlsdorff et al. supplementary material

## Supplementary material

The supplementary material for this article can be found at http://doi.org/10.1017/S0033291725102845.

## References

[r1] Alblooshi, S., Taylor, M., & Gill, N. (2023). Does menopause elevate the risk for developing depression and anxiety? Results from a systematic review. Australasian Psychiatry, 31(2), 165–173. 10.1177/10398562231165439/ASSET/IMAGES/LARGE/10.1177_10398562231165439-FIG1.JPEG. 36961547 PMC10088347

[r2] Badawy, Y., Spector, A., Li, Z., & Desai, R. (2024). The risk of depression in the menopausal stages: A systematic review and meta-analysis. Journal of Affective Disorders, 357, 126–133. 10.1016/j.jad.2024.04.041. 38642901

[r3] Barth, C., Galea, L. A., Jacobs, E. G., Lee, B. H., Westlye, L. T., & Lange, A.-M. G. d. (2025). Menopausal hormone therapy and the female brain: Leveraging neuroimaging and prescription registry data from the UK biobank cohort. eLife, 13. 10.7554/ELIFE.99538.2. PMC12122002 40439116

[r4] Bennett, S., & Mathur, R. (2024). Hormone replacement therapy. Obstetrics, Gynaecology and Reproductive Medicine, 34(3), 58–65. 10.1016/j.ogrm.2023.12.002.

[r5] Bethlehem, R. A. I., Seidlitz, J., White, S. R., Vogel, J. W., Anderson, K. M., Adamson, C., … Alexander-Bloch, A. F. (2022). Brain charts for the human lifespan. Nature, 604(7906), 525–533. 10.1038/S41586-022-04554-Y;TECHMETA. 35388223 PMC9021021

[r6] British Menopause Society. (2021). What is the menopause? Accessed from: http://thebms.org.uk/wp-content/uploads/2023/08/17-BMS-TfC-What-is-the-menopause-AUGUST2023-A.pdf. Accessed on: 21 August 2025.

[r7] Bromberger, J. T., Kravitz, H. M., Chang, Y., Randolph, J. F., Avis, N. E., Gold, E. B., & Matthews, K. A. (2013). Does risk for anxiety increase during the menopausal transition? Study of women’s health across the nation. Menopause, 20(5), 488–495. 10.1097/GME.0B013E3182730599. 23615639 PMC3641149

[r8] Brosch, K., Stein, F., Schmitt, S., Pfarr, J. K., Ringwald, K. G., Thomas-Odenthal, F., … Kircher, T. (2022). Reduced hippocampal gray matter volume is a common feature of patients with major depression, bipolar disorder, and schizophrenia spectrum disorders. Molecular Psychiatry, (10), 4234–4243. 10.1038/s41380-022-01687-4. 35840798 PMC9718668

[r9] Cintron, D., Lipford, M., Larrea-Mantilla, L., Spencer-Bonilla, G., Lloyd, R., Gionfriddo, M. R., … Murad, M. H. (2017). Efficacy of menopausal hormone therapy on sleep quality: Systematic review and meta-analysis. Endocrine, 55(3), 702–711. 10.1007/S12020-016-1072-9/METRICS. 27515805 PMC5509066

[r10] Crandall, C. J., Mehta, J. M., & Manson, J. E. (2023). Management of menopausal symptoms: A review. JAMA, 329(5), 405–420. 10.1001/JAMA.2022.24140. 36749328

[r11] Deeks, A. A. (2003). Psychological aspects of menopause management. Best Practice & Research Clinical Endocrinology & Metabolism, 17(1), 17–31. 10.1016/S1521-690X(02)00077-5. 12763510

[r12] Dratva, J., Real, F. G., Schindler, C., Ackermann-Liebrich, U., Gerbase, M. W., Probst-Hensch, N. M., … Zemp, E. (2009). Is age at menopause increasing across Europe? Results on age at menopause and determinants from two population-based studies. Menopause, 16(2), 385–394. 10.1097/GME.0B013E31818AEFEF. 19034049

[r13] Fischl, B., Van Der Kouwe, A., Destrieux, C., Halgren, E., Ségonne, F., Salat, D. H., … Dale, A. M. (2004). Automatically parcellating the human cerebral cortex. Cerebral Cortex, 14(1), 11–22. 10.1093/CERCOR/BHG087. 14654453

[r14] Gao, X., Geng, T., Jiang, M., Huang, N., Zheng, Y., Belsky, D. W., & Huang, T. (2023). Accelerated biological aging and risk of depression and anxiety: Evidence from 424,299 UK biobank participants. Nature Communications, 14(1), 1–12. 10.1038/S41467-023-38013-7;TECHMETA. PMC10119095 37080981

[r15] Georgakis, M. K., Kalogirou, E. I., Diamantaras, A. A., Daskalopoulou, S. S., Munro, C. A., Lyketsos, C. G., … Petridou, E. T. (2016). Age at menopause and duration of reproductive period in association with dementia and cognitive function: A systematic review and meta-analysis. Psychoneuroendocrinology, 73, 224–243. 10.1016/J.PSYNEUEN.2016.08.003. 27543884

[r16] Gervais, N. J., Gravelsins, L., Brown, A., Reuben, R., Perovic, M., Karkaby, L., … Einstein, G. (2023). Disturbed sleep is associated with reduced verbal episodic memory and entorhinal cortex volume in younger middle-aged women with risk-reducing early ovarian removal. Frontiers in Endocrinology, 14, 1265470. 10.3389/FENDO.2023.1265470. 37859979 PMC10584319

[r17] Gilsanz, P., Lee, C., Corrada, M. M., Kawas, C. H., Quesenberry, C. P., & Whitmer, R. A. (2019). Reproductive period and risk of dementia in a diverse cohort of health care members. Neurology, 92(17), E2005–E2014. 10.1212/WNL.0000000000007326. 30923235 PMC6511081

[r18] Glasser, M. F., Coalson, T. S., Robinson, E. C., Hacker, C. D., Harwell, J., Yacoub, E., … Van Essen, D. C. (2016). A multi-modal parcellation of human cerebral cortex. Nature, 536(7615), 171. 10.1038/NATURE18933. 27437579 PMC4990127

[r19] Gleason, C. E., Dowling, N. M., Wharton, W., Manson, J. A. E., Miller, V. M., Atwood, C. S., … Asthana, S. (2015). Effects of hormone therapy on cognition and mood in recently postmenopausal women: Findings from the randomized, controlled KEEPS–cognitive and affective study. PLoS Medicine, 12(6), e1001833. 10.1371/JOURNAL.PMED.1001833. 26035291 PMC4452757

[r20] Gold, E. B., Crawford, S. L., Avis, N. E., Crandall, C. J., Matthews, K. A., Waetjen, L. E., … Harlow, S. D. (2013). Factors related to age at natural menopause: Longitudinal analyses from SWAN. American Journal of Epidemiology, 178(1), 70–83. 10.1093/AJE/KWS421. 23788671 PMC3698989

[r21] Greendale, G. A., Karlamangla, A. S., & Maki, P. M. (2020). The menopause transition and cognition. JAMA, 323(15), 1495–1496. 10.1001/JAMA.2020.1757 32163094

[r22] Greendale, G. A., Reboussin, B. A., Hogan, P., Barnabei, V. M., Shumaker, S., Johnson, S., & Barrett-Connor, E. (1998). Symptom relief and side effects of postmenopausal hormones: Results from the postmenopausal estrogen/progestin interventions trial. Obstetrics and Gynecology, 92(6), 982–988. 10.1016/S0029-7844(98)00305-6. 9840563

[r23] Hickey, M., Hueg, T. K., Priskorn, L., Uldbjerg, C. S., Beck, A. L., Anstey, K. J., … Bräuner, E. V. (2023). Depression in mid- and later-life and risk of dementia in women: A prospective study within the Danish nurses cohort. Journal of Alzheimer’s Disease: JAD, 93(2), 779–789. 10.3233/JAD-230091. 37092227

[r24] Huang, D. R., Goodship, A., Webber, I., Alaa, A., Sasco, E. R., Hayhoe, B., & El-Osta, A. (2023). Experience and severity of menopause symptoms and effects on health-seeking behaviours: A cross-sectional online survey of community dwelling adults in the United Kingdom. BMC Women’s Health, 23(1). 10.1186/S12905-023-02506-W. PMC10347781 37452317

[r25] Huh, H., Kim, M., Jung, S., Cho, J. M., Kim, S. G., Park, S., … Cho, S. (2024). Menopausal hormone therapy and risk for dementia in women with CKD: A nationwide observational cohort study. Nephrology, 29(3), 126–134. 10.1111/NEP.14260. 38092706

[r26] Joffe, H., Guthrie, K. A., LaCroix, A. Z., Reed, S. D., Ensrud, K. E., Manson, J. A. E., … Cohen, L. (2014). Randomized controlled trial of low-dose Estradiol and the SNRI venlafaxine for vasomotor symptoms. JAMA Internal Medicine, 174(7), 1058. 10.1001/JAMAINTERNMED.2014.1891. 24861828 PMC4179877

[r27] Kim, H., Yoo, J., Han, K., Lee, D. Y., Fava, M., Mischoulon, D., & Jeon, H. J. (2022). Hormone therapy and the decreased risk of dementia in women with depression: A population-based cohort study. Alzheimer’s Research and Therapy, 14(1). 10.1186/S13195-022-01026-3. PMC9202170 35710453

[r28] Leblanc, E. S., Janowsky, J., Chan, B. K. S., & Nelson, H. D. (2001). Hormone replacement therapy and cognition: Systematic review and meta-analysis. JAMA, 285(11), 1489–1499. 10.1001/JAMA.285.11.1489. 11255426

[r29] Lenth, R., Singmann, H., Love, J., Buerkner, P., & Herve, M. (2018). Package “Emmeans” estimated marginal means, aka least-squares means (R package Version: 1.8. 8).

[r30] Li, Y., Zhao, D., Lv, G., Mao, C., Zhang, Y., Xie, Z., & Li, P. (2021). Individual and additive-effect relationships of sleep problems and severe menopausal symptoms among women in menopausal transition. Menopause, 28(5), 517–528. 10.1097/GME.0000000000001726. 33438893

[r31] Monteleone, P., Mascagni, G., Giannini, A., Genazzani, A. R., & Simoncini, T. (2018). Symptoms of menopause – global prevalence, physiology and implications. Nature Reviews Endocrinology, 14(4), 199–215. 10.1038/nrendo.2017.180 29393299

[r32] NICE. (2015). Diagnosis of menopause and perimenopause | Diagnosis | Menopause | CKS | NICE. https://cks.nice.org.uk/topics/menopause/diagnosis/diagnosis-of-menopause-perimenopause/

[r33] Parish, S. J., Faubion, S. S., Weinberg, M., Bernick, B., & Mirkin, S. (2019). The MATE survey: Men’s perceptions and attitudes towards menopause and their role in partners’ menopausal transition. Menopause, 26(10), 1110. 10.1097/GME.0000000000001373. 31188286 PMC6791510

[r34] Pinheiro, J., Bates, D., DebRoy, S., Sarkar, D., Authors, E., Heisterkamp, S., … R-core. (2021). Linear and nonlinear mixed effects models contact. Linear and Nonlinear Mixed Effects Models, Version, 3(1). Accessed from: https://cran.r-project.org/web/packages/nlme/nlme.pdf. Accessed on 21 August 2025.

[r35] Porter, M., Penney, G. C., Russell, D., Russell, E., & Templeton, A. (1996). A population based survey of women’s experience of the menopause. BJOG: An International Journal of Obstetrics & Gynaecology, 103(10), 1025–1028. 10.1111/J.1471-0528.1996.TB09555.X. 8863703

[r36] Pourhadi, N., Mørch, L. S., Holm, E. A., Torp-Pedersen, C., & Meaidi, A. (2023). Menopausal hormone therapy and dementia: Nationwide, nested case-control study. BMJ, 381. 10.1136/BMJ-2022-072770. PMC10302215 37380194

[r37] Public Health England. (2023). Hundreds of thousands of women experiencing menopause symptoms to get cheaper HRT. Accessed from: https://www.gov.uk/government/news/hundredsof-thousands-of-women-experiencing-menopause-symptoms-to-get-cheaper-hormone-replacement-therapy#:~:text=From 1 April 2023, women,charges (currently £18.70). Accessed on 21 August 2025.

[r38] Riemann, D., Voderholzer, U., Spiegelhalder, K., Hornyak, M., Buysse, D. J., Nissen, C., … Feige, B. (2007). Chronic insomnia and MRI-measured hippocampal volumes: A pilot study. Sleep, 30(8), 955–958. 10.1093/SLEEP/30.8.955. 17702263 PMC1978381

[r39] Saleh, R. N. M., Hornberger, M., Ritchie, C. W., & Minihane, A. M. (2023). Hormone replacement therapy is associated with improved cognition and larger brain volumes in at-risk APOE4 women: Results from the European prevention of Alzheimer’s disease (EPAD) cohort. Alzheimer’s Research and Therapy, 15(1), 1–13. 10.1186/S13195-022-01121-5/FIGURES/3. PMC9830747 36624497

[r40] Schaafsma, M., Homewood, J., & Taylor, A. (2010). Subjective cognitive complaints at menopause associated with declines in performance of verbal memory and attentional processes. Climacteric, 13(1), 84–98. 10.3109/13697130903009187;JOURNAL:JOURNAL:ICMT20;WGROUP:STRING:PUBLICATION. 19722118

[r41] Shen, C., Rolls, E. T., Cheng, W., Kang, J., Dong, G., Xie, C., … Feng, J. (2022). Associations of social isolation and loneliness with later dementia. Neurology, 99(2), E164–E175. 10.1212/WNL.0000000000200583. 35676089

[r42] Shi, Y., Chen, L., Chen, T., Li, L., Dai, J., Lui, S., … Gong, Q. (2017). A meta-analysis of voxel-based brain morphometry studies in obstructive sleep Apnea. Scientific Reports, 7(1), 1–13. 10.1038/s41598-017-09319-6. 28855654 PMC5577238

[r43] Shumaker, S. A., Legault, C., Rapp, S. R., Thal, L., Wallace, R. B., Ockene, J. K., … Wactawski-Wende, J. (2003). Estrogen plus progestin and the incidence of dementia and mild cognitive impairment in postmenopausal women: The women’s health initiative memory study: A randomized controlled trial. JAMA, 289(20), 2651–2662. 10.1001/JAMA.289.20.2651. 12771112

[r44] Steventon, J. J., Lancaster, T. M., Baker, E., Bracher-Smith, M., Escott-Price, V., Ruth, K. S., … Murphy, K. (2023). Menopause age, reproductive span and hormone therapy duration predict the volume of medial temporal lobe brain structures in postmenopausal women. Psychoneuroendocrinology, 106393. 10.1016/J.PSYNEUEN.2023.106393.37774659

[r45] Sung, Y. F., Tsai, C. T., Kuo, C. Y., Lee, J. T., Chou, C. H., Chen, Y. C., … Sun, C. A. (2022). Use of hormone replacement therapy and risk of dementia: A Nationwide cohort study. Neurology, 99(17), E1835–E1842. 10.1212/WNL.0000000000200960. 36240091

[r46] Tandon, V., Sharma, S., Mahajan, A., Mahajan, A., & Tandon, A. (2022). Menopause and sleep disorders. Journal of Mid-Life Health, 13(1), 26–33. 10.4103/JMH.JMH_18_22. 35707298 PMC9190958

[r47] Than, S., Moran, C., Beare, R., Vincent, A. J., Collyer, T. A., Wang, W., … Srikanth, V. K. (2021). Interactions between age, sex, menopause, and brain structure at midlife: A UK biobank study. The Journal of Clinical Endocrinology & Metabolism, 106(2), 410–420. 10.1210/CLINEM/DGAA847. 33205159

[r48] Toffol, E., Heikinheimo, O., & Partonen, T. (2013). Associations between psychological well-being, mental health, and hormone therapy in perimenopausal and postmenopausal women: Results of two population-based studies. Menopause, 20(6), 667–676. 10.1097/GME.0B013E318278EEC1. 23277355

[r49] Van De Mortel, L. A., Thomas, R. M., & Van Wingen, G. A. (2021). Grey matter loss at different stages of cognitive decline: A role for the thalamus in developing Alzheimer’s disease. Journal of Alzheimer’s Disease, 83(2), 705. 10.3233/JAD-210173. PMC8543264 34366336

[r50] Van Essen, D. C., Glasser, M. F., Dierker, D. L., Harwell, J., & Coalson, T. (2012). Parcellations and hemispheric asymmetries of human cerebral cortex analyzed on surface-based atlases. Cerebral Cortex, 22(10), 2241–2262. 10.1093/CERCOR/BHR291. 22047963 PMC3432236

[r51] Van Tol, M. J., Van Der Wee, N. J. A., Van Den Heuvel, O. A., Nielen, M. M. A., Demenescu, L. R., Aleman, A., … Veltman, D. J. (2010). Regional brain volume in depression and anxiety disorders. Archives of General Psychiatry, 67(10), 1002–1011. 10.1001/ARCHGENPSYCHIATRY.2010.121. 20921116

[r52] Warrier, V., Stauffer, E. M., Huang, Q. Q., Wigdor, E. M., Slob, E. A. W., Seidlitz, J., … Bethlehem, R. A. I. (2023). Genetic insights into human cortical organization and development through genome-wide analyses of 2,347 neuroimaging phenotypes. Nature Genetics, 55(9), 1483. 10.1038/S41588-023-01475-Y. 37592024 PMC10600728

[r53] Webb, C. A., Weber, M., Mundy, E. A., & Killgore, W. D. S. (2014). Reduced gray matter volume in the anterior cingulate, orbitofrontal cortex and thalamus as a function of mild depressive symptoms: A voxel-based morphometric analysis. Psychological Medicine, 44(13), 2833. 10.1017/S0033291714000348. 25066703 PMC4280261

[r54] Wium-Andersen, M. K., Jørgensen, T. S. H., Halvorsen, A. H., Hartsteen, B. H., Jørgensen, M. B., & Osler, M. (2022). Association of Hormone Therapy with Depression during Menopause in a cohort of Danish women. JAMA Network Open, 5(11), e2239491–e2239491. 10.1001/JAMANETWORKOPEN.2022.39491. 36318208 PMC9627415

[r55] World Health Organisation. (2025). Mental disorders.

