# Supplementary material for: Emotional and cognitive effects of menopause and hormone replacement therapy
Source: Psychol Med. 2026 Jan 27;56:e24. doi: 10.1017/S0033291725102845 (PMC12885352; doi:10.1017/S0033291725102845)
Supplement: Zuhlsdorff et al. supplementary material [file S0033291725102845sup001.docx]

Supplementary materials

**Results**

| Characteristic  Mean (SD)/N(%) | Total (N=124,278) | Pre-menopausal group (N=49,606) | Post-menopausal non-HRT group (N=52,001) | Post-menopausal HRT group (N=22,671) | P value |
| --- | --- | --- | --- | --- | --- |
| Age | 53.69 (8.05) | 45.88 (3.76) | 58.21 (5.45) | 60.41 (5.54) | p<.0001 |
| Age started menopause | NA | NA | 50.41 (4.05) | 47.26 (5.26) | NA |
| Age started HRT | NA | NA | NA | 49.03 (5.04) | NA |
| BMI | 26.49 (4.96) | 26.21 (5.05) | 27.77 (5.01) | 26.46 (4.60) | p<.0001 |
| Higher education | 53,065 (42.7%) | 22,607 (45.6%) | 21,647 (41.6%) | 8,811 (38.9%) | p<.0001 |
| Annual household income >£30,999 | 71,993 (57.9%) | 35,034 (70.6%) | 26,555 (51.07%) | 10,344 (45.6%) | p<.0001 |
| Current smoker | 10,022 (8.06%) | 4,715 (9.50%) | 3,456 (6.64%) | 1,851 (8.16%) | p<.0001 |
| Antidepressant/ anxiolytic use | 2,643 (2.13%) | 1,400 (2.82%) | 833 (1.60%) | 410 (1.81%) | p<.0001 |

**Table S1.** Participant characteristics of sample used for reaction time task analyses.

| Characteristic  Mean (SD)/N(%) | Total (N=43,579) | Pre-menopausal group (N=16,685) | Post-menopausal non-HRT group (N=19,158) | Post-menopausal HRT group (N=7,736) | P value |
| --- | --- | --- | --- | --- | --- |
| Age | 53.99 (8.13) | 45.83 (3.88) | 58.37 (5.40) | 60.80 (5.50) | p<.0001 |
| Age started menopause | NA | NA | 50.52 (4.04) | 47.41 (5.29) | NA |
| Age started HRT | NA | NA | NA | 49.07 (5.08) | NA |
| BMI | 26.52 (5.01) | 26.22 (5.06) | 27.77 (5.01) | 26.46 (4.60) | p<.0001 |
| Higher education | 18,720 (43.0%) | 7,620 (45.7%) | 8,060 (42.1%) | 3,541 (45.8%) | p<.0001 |
| Annual household income >£30,999 | 25,080 (57.6%) | 11,658 (69.9%) | 9,881 (51.57%) | 3,541 (45.8%) | p<.0001 |
| Current smoker | 3,392 (7.78%) | 1,570 (9.41%) | 1,204 (6.28%) | 1,854 (7.99%) | p<.0001 |
| Antidepressant/ anxiolytic use | 1,014 (2.33%) | 537 (3.22%) | 327 (1.71%) | 150 (1.94%) | p<.0001 |

**Table S2.** Participant characteristics of sample used for prospective memory analyses.

| Characteristic  Mean (SD)/N(%) | Total (N=13,144) | Pre-menopausal group (N=5,308) | Post-menopausal non-HRT group (N=5,515) | Post-menopausal HRT group (N=2,321) | P value |
| --- | --- | --- | --- | --- | --- |
| Age | 53.52 (8.26) | 45.56 (4.02) | 58.28 (5.47) | 60.42 (5.55) | p<.0001 |
| Age started menopause | NA | NA | 50.51 (4.03) | 47.06 (5.46) | NA |
| Age started HRT | NA | NA | NA | 48.74 (5.22) | NA |
| BMI | 26.50 (4.97) | 26.33 (5.07) | 26.68 (4.97) | 26.46 (4.69) | p<.0001 |
| Higher education | 5,260 (40.0%) | 2,247 (42.3%) | 2,183 (39.6%) | 830 (35.8%) | p<.0001 |
| Annual household income >£30,999 | 7,253 (55.2%) | 3,622 (68.2%) | 2,668 (48.38%) | 963 (41.5%) | p<.0001 |
| Current smoker | 1,056 (8.03%) | 521 (9.82%) | 346 (6.72%) | 189 (8.14%) | p<.0001 |
| Antidepressant/ anxiolytic use | 328 (2.50%) | 171 (3.22%) | 104 (1.89%) | 53 (2.28%) | p<.0001 |

**Table S3.** Participant characteristics of sample used for digit span task analyses.

| Characteristic  Mean (SD)/N(%) | Total (N=10,873) | Pre-menopausal group (N=762) | Post-menopausal non-HRT group (N=7,583) | Post-menopausal HRT group (N=2,528) | P value |
| --- | --- | --- | --- | --- | --- |
| Age | 63.53 (7.22) | 52.28 (3.32) | 63.65 (6.45) | 66.58 (6.98) | p<.0001 |
| Age started menopause | NA | NA | 51.04 (3.99) | 47.88 (5.34) | NA |
| Age started HRT | NA | NA | NA | 49.88 (5.72) | NA |
| BMI | 25.82 (4.62) | 26.15 (4.92) | 25.84 (4.67) | 25.65 (4.36) | p<.0001 |
| Higher education | 5,389 (49.6%) | 424 (55.6%) | 3,758 (49.6%) | 1,207 (47.7%) | p<.0001 |
| Annual household income >£30,999 | 5,657 (52.0%) | 544 (71.4%) | 3,844 (50.69%) | 1,269 (50.2%) | p<.0001 |
| Current smoker | 294 (2.70%) | 38 (4.99%) | 189 (2.49%) | 67 (2.65%) | p<.0001 |
| Antidepressant/ anxiolytic use | 223 (2.05%) | 24 (3.15%) | 137 (1.81%) | 62 (2.45%) | p<.0001 |

**Table S4.** Participant characteristics of sample used for neuroimaging analyses.
